# Phenotypic changes associated with Colistin resistance due to Lipopolysaccharide loss in *Acinetobacter baumannii*

**DOI:** 10.1080/21505594.2018.1460187

**Published:** 2018-05-15

**Authors:** Marta Carretero-Ledesma, Meritxell García-Quintanilla, Reyes Martín-Peña, Marina R. Pulido, Jerónimo Pachón, Michael J. McConnell

**Affiliations:** a Clinical Unit of Infectious Diseases, Clinical Microbiology and Preventive Medicine; Institute of Biomedicine of Seville (IBiS), University Hospital Virgen del Rocío/CSIC /University of Seville, Seville, Spain; b Department of Medicine, University of Seville, Seville, Spain

**Keywords:** *Acinetobacter baumannii*, colistin, fitness, lipopolysaccharide, virulence

## Abstract

*Acinetobacter baumannii* can acquire resistance to colistin via complete loss of lipopolysaccharide (LPS) biosynthesis due to mutations in the *lpxA*, *lpxC* and *lpxD* genes. However, although colistin is increasingly being used for the treatment of multidrug resistant infections, very few *A. baumannii* clinical isolates develop colistin resistance through loss of LPS biosynthesis. This may suggest that LPS loss affects virulence traits that play a role in the transmission and pathogenesis of *A. baumannii*. In this study we characterize multiple virulence phenotypes of colistin resistant, LPS-deficient derivatives of the ATCC 19606 strain and five multidrug resistant clinical isolates and their colistin resistant, LPS-deficient derivatives. Our results indicate that LPS loss results in growth defects compared to the parental strain *in vitro* both in laboratory media and human serum (competition indices of 0.58 and 7.0 × 10^−7^, respectively) and reduced ability to grow and disseminate *in vivo* (competition index 6.7 × 10^−8^). Infection with the LPS-deficient strain resulted in lower serum levels of pro-inflammatory cytokines TNF-α and IL-6 compared to the parent strain, and was less virulent in a mouse model of disseminated sepsis. LPS loss also significantly affected biofilm production, surface motility, growth under iron limitation and susceptibility to multiple disinfectants used in the clinical setting. These results demonstrate that LPS loss has a significant effect on multiple virulence traits, and may provide insight into the low incidence of colistin resistant strains lacking LPS that have been reported in the clinical setting.

## Introduction

*Acinetobacter baumannii* is a Gram negative, predominately nosocomial pathogen that produces different types of infections including pneumonia, bloodstream infections and meningitis, among others [1]. Over the preceding two decades, the number of nosocomial infections caused by *A. baumannii* strains that have acquired resistance to multiple antibiotic classes has increased alarmingly, and has limited the use of antibiotics that previously demonstrated activity against circulating strains. This has prompted the increasing use of colistin, often one of the few clinically-used antibiotics that retains activity against many multidrug resistant isolates. Colistin is a peptide antibiotic that targets the bacterial cell membrane through interaction with the lipid A moiety of the lipopolysaccharide (LPS) molecule. Colistin was introduced into clinical use approximately 60 years ago, but its use diminished, until recently, due to concerns over nephrotoxicity and neurotoxicity [2]. Unfortunately, the increased use of colistin in recent years has resulted in the emergence of colistin resistant *A. baumannii* [3–5].

Two mechanisms that confer resistance to colistin in *A. baumannii* have previously been described. Mutations in the PmrAB two component system result in colistin resistance via the addition of phosphoethanolamine to the lipid A component of LPS [6,7]. This modification confers resistance by decreasing the net negative charge of LPS, thus lowering its affinity for positively charged colistin molecules. The second mechanism producing colistin resistance is the complete loss of LPS expression due to mutations in the enzymes involved in its biosynthesis *lpxA*, *lpxC*, and *lpxD*, resulting in a lack of the molecule targeted by colistin [8,9]. LPS is a principal component of the outer face of the bacterial outer membrane in Gram negative bacteria and is responsible for creating a permeability barrier for diffusion of substances into the cell, and maintaining the structural integrity of the bacteria [10]. Early work in *Escherichia coli* and *Salmonella* indicated that LPS biosynthesis was essential for bacterial cell viability [11]. Subsequent studies, however, showed that three bacterial species are viable in the absence of LPS expression, *Neisseria meningitidis*, *Moraxella catarrhalis*, and recently *A. baumannii* [9,12,13].

A handful of studies have characterized the effect of LPS loss in *A. baumannii* on different aspects of bacterial physiology. Previous work by our group has demonstrated that LPS loss in *A. baumannii* produces increased susceptibility to azithromycin, rifampicin and vancomycin, and results in partial colistin dependence, a phenomenon in which the presence of colistin increases the growth rate of LPS-deficient strains [14]. We and others have also shown that LPS loss can alter susceptibility to host antimicrobial peptides [15,16]. Two independent studies have reported the decreased fitness of LPS-deficient *A. baumannii* in animal models of infections [17,18], and a separate study demonstrated reduced toll-like receptor signaling during experimental infection with these strains [16]. Finally, a transcriptomic study comparing gene expression between LPS-deficient and wildtype strains demonstrated increased expression of genes involved in the synthesis and transport of lipoproteins, phospholipids, and poly-β-1,6-N-acetylglucosamine [19], perhaps suggesting a compensatory response to the loss of LPS in the bacterial membrane.

In spite of the high-level resistance to colistin that is conferred by LPS loss, there are very few reports describing infection with LPS-deficient *A. baumannii* in the clinical setting. One possibility is that loss of LPS results in strains with reduced fitness and a lower capacity for surviving in the environment and being transmitted between patients. In this context, our goal in the present study was to characterize changes in virulence traits associated with LPS loss in *A. baumannii* such as in vitro and in vivo fitness, in vivo dissemination, induction of pro-inflammatory cytokines, biofilm production, surface motility, growth under iron limiting conditions and susceptibility to disinfectants commonly used in the clinical setting. Our results indicate that LPS loss affects numerous traits that may be associated with the virulence and transmissibility of *A. baumannii*, which may, at least in part, explain the low incidence of infection with LPS-deficient strains.

## Materials and methods

### Bacterial strains used in this study

The *A. baumannii* ATCC 19606 strain is an antibiotic-susceptible reference strain. Colistin-resistant derivatives of ATCC 19606, IB002, IB003, IB004, IB006, IB007 were selected for by plating ATCC 19606 on Mueller-Hinton plates containing 10 mg/l of colistin, as described previously [9]. The *lpxA*, *lpxC* and *lpxD* genes from the resulting colistin-resistant derivatives were sequenced to identify mutations. Furthermore, five clonally distinct, multidrug resistant clinical isolates Ab-84, Ab-108, Ab-167, Ab-176, Ab-208 that have been previously described [20], and their colistin resistant, LPS deficient derivatives Ab-84R, Ab-108R, Ab-167R, Ab-176R, Ab-208R which were obtained in a previous study by selecting for growth in the presence of colistin were employed [15]. Each of the LPS deficient, colistin resistant derivatives contains a mutation in one of the LPS biosynthesis genes *lpxA*, *lpxC* or *lpxD* (Table 1). The mutants strains were confirmed as colistin resistant by determining colistin MICs by broth microdilution according to Clinical Laboratory Standards Institute guidelines [21]. Loss of LPS was confirmed using the QCL-1000 *Limulus* Amebocyte Assay (Lonza) according to the manufacturer's instructions to quantify endotoxin levels in three independent cultures of each strain. In order to complement the IB004 derivative (*lpxA* mutation), the pWH1266-*lpxA* plasmid was constructed by cloning the wild type *lpxA* reading frame into the BamH1 site of pWH1266 as described previously [9]. The plasmid was introduced into IB004 via electroporation and transformants were selected on 80 mg/l ticarcillin. CS01 is a colistin sensitive, multidrug resistant clinical isolate, and CR17 is its colistin resistant derivative containing a M12K mutation in *pmrA*.

**Table 1.** Colistin MIC values and endotoxin levels of strains used in this study.

Strain	Mutation/Description	Colistin MIC (mg/l)	EU/ 10 [6] cells^a^
ATCC 19606	Antibiotic susceptible reference strain	≤ 0.25	1060 ± 814
IB002	*ISAba11* insertion at nucleotide 372 of *lpxC* gene	> 128	< 1
IB003	*ISAba11* insertion at nucleotide 394 of *lpxC* gene	> 128	< 1
IB004	Deletion of nucleotide 461 of *lpxA* gene generating frameshift after S153	> 128	< 1
IB006	*ISAba11* insertion at nucleotide 393 of *lpxC* gene	> 128	< 1
IB007	*ISAba11* insertion at nucleotide 393 of *lpxC* gene	> 128	< 1
IB004 + pWH1266-*lpxA*	IB004 complemented with the pWH1266 plasmid expressing the wild type *lpxA* gene	≤ 0.25	421 ± 67.0
Ab-84	Multidrug resistant clinical isolate	≤ 0.25	17.78 ± 3.5
Ab-108	Multidrug resistant clinical isolate	≤ 0.25	18.53 ± 4.80
Ab-167	Multidrug resistant clinical isolate	≤ 0.25	20.02 ±2.36
Ab-176	Multidrug resistant clinical isolate	≤ 0.25	42.97 ±21.52
Ab-208	Multidrug resistant clinical isolate	≤ 0.25	25.02±5.04
Ab-84R	Strain Ab-84 containing a 40 nucleotide insertion at nucleotide 321 of *lpxC* generating a premature stop codon	> 128	< 1
Ab-108R	Strain Ab-108 with a T614A mutation in *lpxA* producing an I205N amino acid substitution	> 128	< 1
Ab-167R	Strain Ab-167 containing an *ISAba1* insertion at nucleotide 321 of *lpxC*	> 128	< 1
Ab-176R	Strain Ab-176 with a G739T substitution at nucleotide 739 of the *lpxD* gene producing a premature stop codon	> 128	< 1
Ab-208R	Strain Ab-208with a C593A substitution at nucleotide 198 of the *lpxD* gene producing A198E	>256	< 1
CS01	Colistin susceptible *A. baumannii* clinical isolate	≤ 0.25	ND
CR17	Colistin resistant derivative of CS01 containing a M12K mutation in *pmrA*	64	ND

a Data represent the mean ± the standard error of the mean of three independent assays.

EU; Endotoxin Units.

### In vitro and in vivo growth

For *in vitro* growth curves, bacteria at a concentration of 1 × 10^6^ CFU/ml were grown in 5 ml of Mueller-Hinton broth or inactivated human serum (type AB, Sigma) at 37ºC in static cultures. Aliquots of 100 µl were taken at the indicated time points and dilutions were plated on blood agar for ATCC 19606 and IB004 + pWH1266-*lpxA* strains and Mueller-Hinton plates containing 10 mg/l colistin for IB004 (n = 3 cultures/strain). For *in vivo* growth curves, female C57BL/6 mice (University of Seville) were infected using an intraperitoneal sepsis model that has been described previously [22,23]. This model results in bacterial dissemination to distant organs and typically produces mortality between 24 and 48 hours. Mice were infected with 2.5 × 10^5^ CFU of the ATCC 19606, IB004 and IB004 + pWH1266-*lpxA* strains and spleens, lungs and kidneys were aseptically removed at the indicated time points (n = 4 mice/time point). Tissues were weighed and homogenized for determination of bacterial loads by plating on the appropriate media. All experiments involving the use of animals were approved by the University Hospital Virgen del Rocío Committee on Ethics and Experimentation (Evaluation code: 2013PI/296). In all experiments, efforts were made to minimize suffering, and any animals appearing moribund during the course of experimentation were immediately euthanized.

### *In vitro* and *in vivo* competition indices

Growth in competition between ATCC 19606 and IB004, and, in a separate experiment ATCC 19606 and IB004 + pwH1266-*lpxA* was assessed in Mueller-Hinton broth and human serum by mixing 5.0 × 10^5^ CFU/ml of each strain in the same culture. At 24 hours, aliquots from the cultures were plated on both blood agar plates and Mueller-Hinton plates containing 10 mg/L of colistin in order to determine the number of colonies from each strain (n = 3 cultures/assay). For *in vivo* competition experiments, groups of three C57BL/6 mice were infected with a mixture of 1.25 × 10^5^ CFU/mouse of each strain using the intraperitoneal infection model described above. After 24 hours, spleen homogenates were plated on both blood agar plates and Mueller-Hinton plates containing 10 mg/L of colistin to determine the bacterial load of each strain. Competition indices were defined as the number of CFUs recovered from the mutant strain/the number of CFUs recovered from the ATCC 19606 strain, divided by the number of CFUs in the mutant inoculum/the number of CFUs in the ATCC 19606 inoculum. In cases where no colonies were recovered from cultures or tissues, the limit of detection of the assay was used to calculate competition indices.

### Serum cytokine levels and virulence

Groups of six C57BL/6 mice were infected intraperitoneally with 2.5 × 10^5^ CFU/mouse of the indicated strains, and sera were collected at 12 hours post-infection. TNF-α and IL-6 levels in each sample were determined using the TNF-α and IL-6 OptiEIA Cytokine Sets (BD) as described previously [22]. The ability of each strain to produce mortality in mice was assessed using the mouse model of intraperitoneal sepsis described above. Groups of eight C57BL/6 mice were inoculated intraperitoneally with 2.5 × 10^5^ CFU/mouse and monitored for survival daily for 7 days.

### Bacterial growth rate

Overnight cultures were adjusted to a concentration of 5 × 10^5^ CFU /ml in Mueller-Hinton broth and 200 µl of the bacterial suspensions were added to wells of 96-well flat bottom polystyrene microplates and growth at 37ºC was quantified by measuring the OD_595_ every 30 minutes over 24 hours using an Infinite 200 Pro plate reader (Tecan Trading AG, Switzerland). Doubling times were calculated by determining the time necessary for duplication of the optical density using the exponential phase of the growth curve. All assays were performed in triplicate.

### Biofilm production

Biofilm production was measured based on a previously described method [24]. Strains were grown overnight in Mueller-Hinton II broth and then adjusted to a final concentration of 5 × 10^3^ CFU/ml in Mueller-Hinton II broth on the day of the assay. Two-hundred µl of the bacterial suspensions were placed into wells of a U-shaped polystyrene 96-well plate and statically incubated at 37ºC for 24 hours. After incubation, wells were washed twice by immersion in distilled water, followed by the addition of 200 µl of a 0.4% crystal violet solution. After 10 minutes of incubation in darkness at room temperature the excess crystal violet solution was removed and the plates were washed twice with distilled water and allowed to dry at room temperature. Two-hundred µl of 95% ethanol (v/v) was added to each well and incubated in darkness at room temperature for 10 minutes, followed by reading at an absorbance of 580 nm on a plate reader after transfer of the solution to a clean flat-bottom 96-well plate. All assays were performed in triplicate.

### Surface motility

Overnight cultures of each strain were adjusted to a concentration of 1 × 10^7^ CFU/ml in Mueller-Hinton broth. Two µl of the bacterial suspension were placed in the center of a Luria Bertani plate containing 0.3% agarose. Plates were allowed to dry for 30 minutes in a laminar flow hood and then incubated in at 37ºC with 80% of humidity. The radii of surface extension were measured at 24, 48 and 72 hours of incubation. All assays were performed in triplicate.

### Growth in iron limiting conditions

Overnight cultures were adjusted to a concentration of 5 × 10^5^ CFU /ml in Mueller-Hinton broth with or without the iron chelator 2,2’-bipyridyl (Bip) at a final concentration of 150 µM. Two-hundred µl of the bacterial suspensions were added to wells of 96-well flat bottom polystyrene microplates and growth at 37ºC was quantified by measuring the OD_595_ every 30 minutes over 24 hours using an Infinite 200 Pro plate reader (Tecan Trading AG, Switzerland). All assays were performed in triplicate.

### Susceptibility to disinfectants

Minimum inhibitory concentration (MIC) values were determined for chlorhexidine, deoxycholate, sodium dodecyl sulfate, ethanol and povidone iodine. Overnight cultures of each strain were adjusted to 5 × 10^5^ CFU/ml in Mueller-Hinton broth II and MIC values were determined according the recommendations of the Clinical and Laboratory Standards Institute for antimicrobials [21]. For each strain MIC values were determined on two different days in duplicate.

### Statistical analysis

The distribution of all data sets was assessed using the Shapiro Wilk test. Cytokine levels were compared using a one-way ANOVA, and differences between groups were determined using the Tukey post-hoc test. Survival was compared using the log-rank test. Biofilm production and surface motility were compared using the Student t test for pairwise analyses. Comparison of more than three data sets was performed using a one-way ANOVA. A *p* value ≤ 0.05 was considered significant.

## Results

### Isolation of colistin-resistant, LPS-deficient strains

The method used for selection of colistin resistant strains described in the methodology resulted in the selection of a total of 11 resistant mutants. Of these, five were selected for further study after sequencing of the *lpxA*, *lpxC* and *lpxD* genes revealed mutations that resulted in lack of LPS biosynthesis (Table 1). Four strains had mutations in the *lpxC* gene, all of which were due to insertion of *ISAba11*, and one strain had a single nucleotide deletion in *lpxA*. All derivative of the ATCC 19606 strain were highly resistant to colistin (MIC > 128 mg/L), and showed no endotoxin activity, confirming the loss of LPS production. Complementation of the IB004 strain with a plasmid encoding a wildtype copy of the *lpxA* gene (IB004 + pWH1266-*lpxA*) reverted the phenotype to colistin susceptibility and endotoxin production. As described previously, the colistin resistant derivatives of the multidrug resistant clinical isolates Ab-84, Ab-108, Ab-167, Ab-176 and Ab-208, demonstrated high level resistance to colistin and the absence of endotoxin activity (Table 1). On the basis that all mutant strains were phenotypically similar with respect to colistin resistance and LPS loss, IB004 and its complemented counterpart (IB004 + pWH1266-*lpxA*) were selected for further analysis for in vitro and in vitro growth and fitness studies.

### In vitro and in vivo growth of LPS-deficient *A. baumannii*

**Figure 1.**
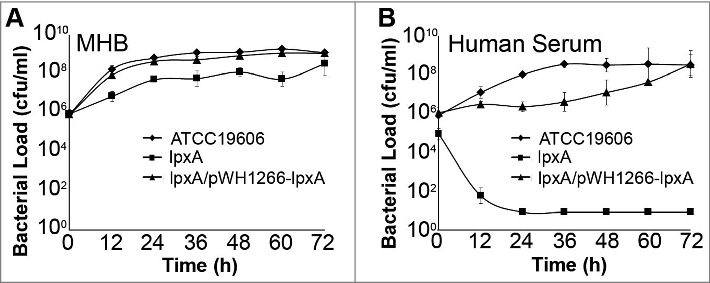
*In vitro* growth of LPS-deficient *A.* *baumannii*. Graphs represent the growth of ATCC 19606, IB004 and IB004 + pWH1266-*lpxA* in Mueller Hinton broth (A) and human serum (B). Data points represent the average of three independent cultures with error bars representing the standard deviation.

The effect of LPS loss on bacterial growth in rich media (Mueller Hinton broth) was assessed. IB004 was able to replicate in Mueller-Hinton broth, but showed a reproducible decrease in growth compared to the ATCC 19606 and IB004 + pWH1266-*lpxA* strains of approximately 1 log_10_ at all time points (Fig. 1A). In human serum, however, IB004 showed a marked growth defect when compared to the ATCC 19606 and IB004 + pWH1266-*lpxA* strains, with bacteria present only at the 0 and 12 hour time points, and no detectable bacteria beyond 24 hours (Fig. 1B). The difference in growth between rich media and human serum indicate that under laboratory conditions a modest decrease in fitness is observed, whereas conditions similar to those encountered by *A. baumannii* during human infection result in a dramatic fitness loss.

**Figure 2.**
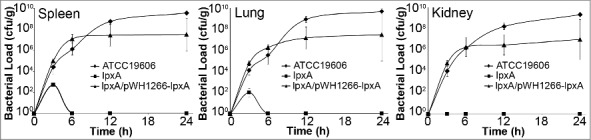
Growth and dissemination of LPS-deficient *A.* *baumannii* in an experimental mouse model. Tissue bacterial loads of ATCC 19606, IB004 and IB004 + pWH1266-*lpxA* in spleen, lungs and kidneys of mice infected with 2.5 × 10^5^ CFU of each strain (n = 4 mice/timepoint). Data points represent the average independent mice with error bars representing the standard deviation.

The loss of LPS also dramatically affected the ability of IB004 to survive and replicate *in vivo* in a mouse model of disseminated sepsis. In this model, intraperitoneal administration of *A. baumannii* results in rapid bacterial dissemination to distant organs and typically produces death within 48 hours. After infection with the IB004, low bacterial loads (approximately 10^2^ CFU/g) were seen in spleens and lungs only at three hours post-infection, and no bacteria were detected in kidneys at any time point (Fig. 2). Conversely, ATCC 19606 and IB004 + pWH1266-*lpxA* were able to grow and replicate in all organs tested. Taken together, these data indicate that LPS loss results in an inability to replicate under physiological conditions and produce disseminated infection.

### Fitness loss of LPS-deficient *A. baumannii*

**Figure 3.**
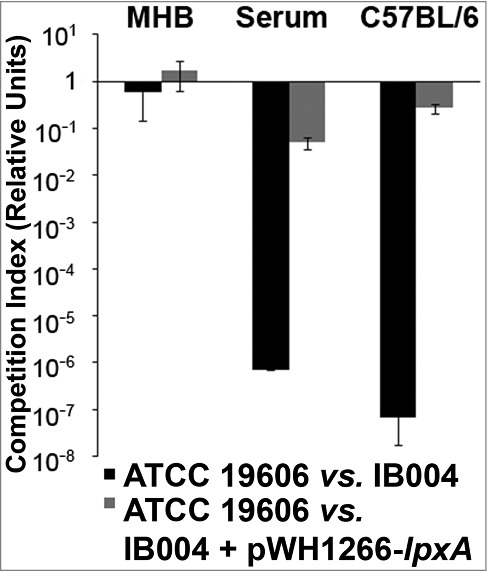
Fitness of LPS-deficient *A.* *baumannii* with its wild type counterpart in laboratory medium, human serum and mouse tissue. Competition indices comparing ATCC 19606 and IB004, and ATCC 19606 and IB004 + pWH1266-*lpxA* in Muller Hinton broth, human serum and mouse spleen. Bars represent the average of three independent assays with error bars representing the standard deviation.

In order to quantify the decrease in fitness resulting from LPS loss, competition indices were determined at 24 hours in Mueller Hinton broth, human serum and *in vivo* by directly comparing the growth of the IB004 and IB004 + pWH1266-*lpxA* to ATCC 19606 when grown together (Fig. 3). In Mueller Hinton broth IB004 demonstrated a modest loss of fitness compared to the wild type strain (competition index; 0.58), whereas in human serum and *in vivo* a dramatic fitness loss was observed (competition indices; 7.1 × 10^−7^ and 6.7 × 10^−8^, respectively). These results are in agreement with the growth patterns observed in Figs. 1 and 2. Interestingly, the IB004 + pWH1266-*lpxA* strain also demonstrated a moderate loss of fitness in human serum and *in vivo* compared to the wild type strain (5 × 10^−2^ and 2.7 × 10^−1^, respectively), which may be due to the increased energy requirements associated with the maintenance of the complementing plasmid.

### Cytokine response to infection with LPS-deficient *A. baumannii*

**Figure 4.**
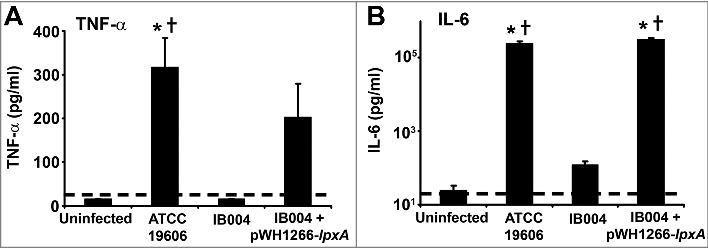
Serum cytokine levels after infection with LPS-deficient *A.* *baumannii* in a mouse model of experimental infection. Serum TNF-α (A) and IL-6 (B) levels in uninfected, ATCC 19606 infected, IB004-infected and IB004 + pWH1266-*lpxA*-infected mice (n = 6 mice/group) 12 hours after infection with 2.5 × 10^5^ CFU of the indicated strain. Bars represent average values and error bars represent the standard deviation. *P < 0.01 compared to uninfected mice, † *P* < 0.01 compared to IB004-infected mice; Tukey post-hoc test.

In order to evaluate the effect of LPS loss on the virulence of *A. baumannii* during infection, post-infection pro-inflammatory cytokine levels in serum and mortality were determined for each strain in a mouse model of disseminated sepsis which was previously shown to induce elevated serum TNF-α and IL-6 levels [22]. After infection with the IB004 strain, TNF-α levels were below the limit of detection in all mice, similar to results obtained in uninfected mice (Fig. 4A). In contrast, mice infected with the wild type strain had detectable levels of TNF-α which were significantly higher than levels in uninfected and IB004-infected mice (*P* < 0.01; Tukey post-hoc test). TNF-α levels in mice infected with IB004 + pWH1266-*lpxA* were not significantly different than in mice infected with the ATCC 19606 strain (*P* = 0.473; Tukey post-hoc test). IL-6 levels were significantly higher in ATCC 19606 and IB004 + pWH1266-*lpxA* infected mice than in mice infected with the IB004 strain and uninfected mice (*P* < 0.01; Tukey post-hoc test; Fig. 4B).

### Virulence of LPS-deficient *A. baumannii* in an experimental mouse model

**Figure 5.**
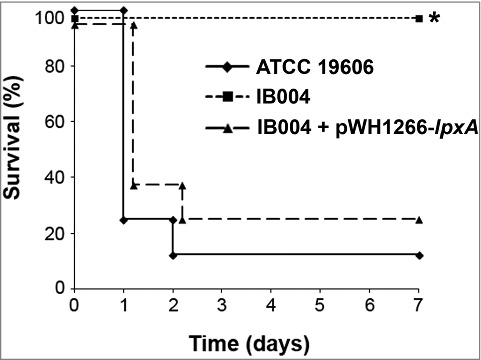
Virulence of LPS-deficient *A.* *baumannii* in a mouse model of disseminated infection. Survival of mice infected with 2.5 × 10^5^ CFU the indicated strains in a disseminated sepsis model during 7 days post-infection. *P < 0.005, log-rank test compared to ATCC 19606 and IB004 + pWH1266-*lpxA* infected mice.

To assess virulence, mice were infected with 2.5 × 10^5^ CFU of each strain and mortality was monitored over 7 days. This inoculum represents 39.6 times the LD_50_ of the ATCC 19606 strain, an inoculum which has previously been shown to be lethal in this animal model [22]. As can be seen in Fig. 5, significant mortality was seen in the ATCC 19606 and IB004 + pWH1266-*lpxA* -infected mice (87.5% and 75.0%, respectively), whereas no mice in the IB004-infected group died (*P* < 0.005 compared to both groups; log-rank test; Fig. 5).

These results indicated that loss of LPS biosynthesis resulted in decreased virulence in a mouse model. We next wanted to characterize how LPS loss affected individual traits that could be associated with bacterial virulence or transmission. To this end, the ATCC 19606 strain and IB004, as well as five multidrug resistant clinical isolates and their LPS-deficient derivatives, were assessed with respect to growth rate, biofilm formation, surface motility, growth under iron limitation and disinfectant susceptibility.

### Effect of LPS loss on growth rate and biofilm production

**Figure 6.**
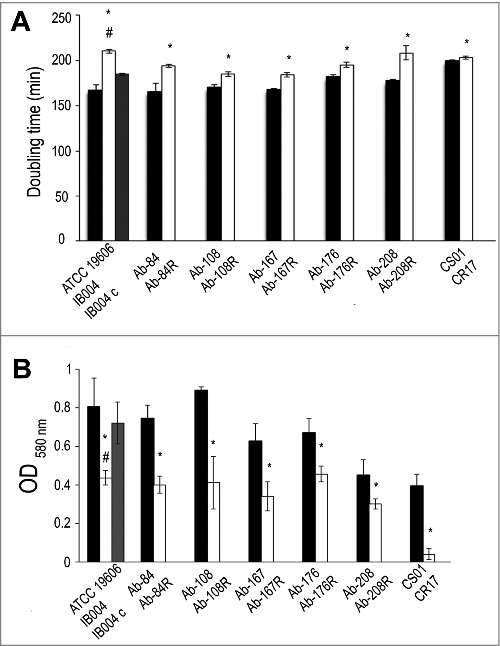
Effect of LPS loss on growth rate and biofilm production. Growth rate (A) and biofilm production (B) were determined for ATCC 19606, IB004, and IB004 + pWH1266-*lpxA* (IB004 C), and 5 pairs of multidrug resistant clinical isolates (Ab-84, Ab-108, Ab-167, Ab-178 and Ab-208) and their LPS-deficient derivatives (Ab-84R, Ab-108R, Ab-167R, Ab-178R and Ab-208R) and CS01/CR17. Bars represent the average of three separate assays, with error bars representing the standard deviation. *P < 0.005 compared to parental, LPS-replete strain, Student's t test. # *P* < 0.005 compared to complemented strain, Student's t test.

In order to assess the effect of LPS loss on growth rate, doubling times were determined for the ATCC 19606 strain and its LPS-deficient counterpart, IB004, were assessed. As can be seen in Fig. 6A, IB004 grew significantly slower than the parent strain. Complementation of the IB004 strain with a wildtype copy of the *lpxA* gene (IB004 + pWH1266-*lpxA)* partially restored growth rate to almost wildtype levels, indicating that decreased growth was due to LPS loss. Doubling times were also determined for five multidrug resistant clinical isolates and their LPS-deficient derivatives. In all cases, the LPS-deficient derivatives grew significantly slower than their wildtype counterparts (Fig. 6A). The CR17 strain, which contains a mutation in the PmrAB system also showed reduced growth compared to its colistin susceptible counterpart (CS01).

Biofilm production in the ATCC 19606 strain and its LPS-deficient counterpart, IB004, were assessed. As can be seen in Fig. 6B, IB004 produced significantly less biofilm after 24 hours than the parent strain. Complementation of the IB004 strain with a wildtype copy of the *lpxA* gene (IB004 + pWH1266-*lpxA)* restored biofilm production to almost wildtype levels, indicating that the decreased biofilm production was due to LPS loss. Biofilm production was also determined for five multidrug resistant clinical isolates and their LPS-deficient derivatives. In all cases, the LPS-deficient derivatives produced significantly less biofilm than their wildtype counterparts (Fig. 6B). The CR17 strain, which contains a mutation in the PmrAB system, also demonstrated reduced biofilm formation compared to its colistin susceptible counterpart (CS01).

### Effect of LPS loss on surface motility

**Figure 7.**
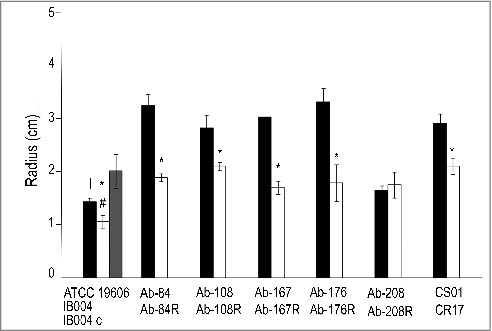
Effect of LPS loss on surface motility. Surface motility on semisolid media of ATCC 19606, IB004, and IB004 + pWH1266-*lpxA*, and 5 pairs of multidrug resistant clinical isolates (Ab-84, Ab-108, Ab-167, Ab-178 and Ab-208) and their LPS-deficient derivatives (Ab-84R, Ab-108R, Ab-167R, Ab-178R and Ab-208R) and CS01/CR17 after 72 hours of incubation. Bars represent the average of three separate assays, with error bars representing the standard deviation. *P < 0.005 compared to parental, LPS-replete strain, Student's t test. # *P* < 0.005 compared to complemented strain, Student's t test (A). Surface motility of the Ab-176 strain and its LPS-deficient counterpart after 72 hours of incubation (B).

Surface motility of the ATCC 19606 strain and its LPS-deficient counterpart, IB004, were assessed. As shown in Fig. 7A, IB004 showed reduced surface motility compared to ATCC 19606. Complementation of IB004 resulted in restoration of surface motility. Surface motility was also characterized in the five multidrug resistant clinical isolates and their LPS-deficient derivatives (Fig. 7A and B). For Ab-84, Ab-108, Ab-167 and Ab-176, LPS loss resulted in a significant reduction in surface motility. In strain Ab-208, no significant changes were observed upon LPS loss, indicating that the effects of LPS loss on surface motility may be strain specific. The CR17 strain, which contains a mutation in the PmrAB system, also demonstrated reduced surface motility compared to its colistin susceptible counterpart (CS01).

### Effect of LPS loss on growth in iron limiting conditions

**Figure 8.**
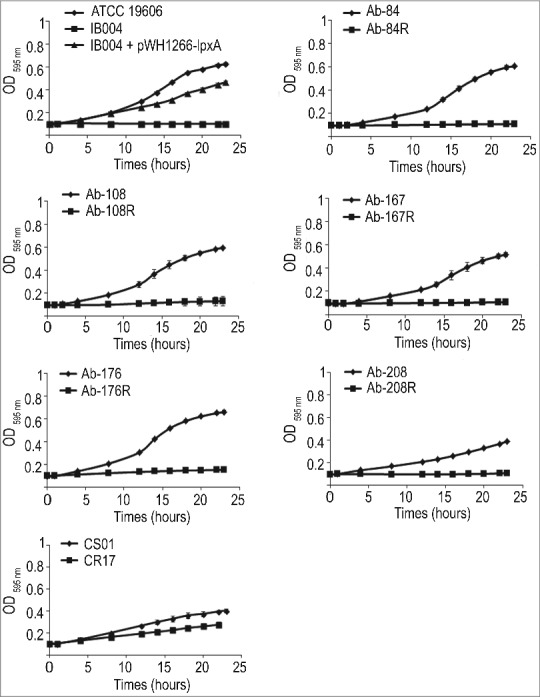
Effect of LPS loss on growth under iron limitation. Growth in iron limiting conditions of ATCC 19606, IB004, and IB004 + pWH1266-*lpxA*, and 5 pairs of multidrug resistant clinical isolates (Ab-84, Ab-108, Ab-167, Ab-178 and Ab-208) and their LPS-deficient derivatives (Ab-84R, Ab-108R, Ab-167R, Ab-178R and Ab-208R) and CS01/CR17 over 24 hours. Bars represent the average of three separate cultures, with error bars representing the standard deviation.

Growth in iron limiting conditions was characterized for the ATCC 19606 strain in its LPS-deficient counterpart, IB004. As can be seen in Fig. 8, loss of LPS resulted in no growth over 24 hours under the conditions tested. However, complementation of the IB004 strain restored growth under iron limitation to near wildtype levels. LPS loss in five multidrug resistant clinical isolates also resulted in strains that were unable to grow under iron limitation (Fig. 8), suggesting that LPS may play a key role in survival in low iron conditions. The CR17 strain, which contains a mutation in the PmrAB system, also demonstrated reduced growth under iron limitation compared to its colistin susceptible counterpart (CS01), although the difference was not as marked compared to strains lacking LPS.

### Effect of LPS loss on susceptibility to disinfectants

Susceptibilities to the disinfectants chlorhexidine, deoxycholate, sodium dodecyl sulfate, ethanol and povidone iodine were determined for the ATCC 19606 strain and its LPS-deficient derivative, IB004. As demonstrated in Table 2, IB004 demonstrated increased susceptibility to chlorhexidine (4-fold), deoxycholate (> 32-fold), sodium dodecyl sulfate (16-fold), and ethanol (2-fold). LPS loss did not affect susceptibility to povidone iodine (Table 2). Complementation of IB004 restored susceptibilities to wildtype levels for all disinfectants. LPS loss in multidrug resistant clinical isolates produced similar results, with increased susceptibility to chlorhexidine, deoxycholate and sodium dodecyl sulfate observed in all strains. Increased susceptibility to ethanol was observed in 2 of 5 strains. Susceptibility to povidone iodine was unchanged in all strains. Importantly, the CR17 strain, which contains a mutation in the PmrAB system, did not show increased susceptibility to disinfectants compared to its colistin susceptible counterpart (CS01).

**Table 2.** Susceptibility (MIC) of *A. baumannii* strains to disinfectants.

Strain	Chlorhexidine (mg/l)	Deoxycholate (mM)	SDS (g/l)	Ethanol (%)	Povidone iodine (g/l)
ATCC 19606	0.487	> 10	1.56	25	6.25
IB004	0.122	0.3125	0.1	12.5	6.25
IB004 + pWH1266-*lpxA*	0.487	> 10	1.56	50	6.25
84S	0.975	> 10	1.56	6.25	6.25
84R	0.061	0.3125	0.1	12.5	6.25
108S	0.487	> 10	6.25	6.25	6.25
108R	0.244	0.625	0.2	6.25	6.25
167S	0.975	> 10	1.56	6.25	6.25
167R	0.122	0.3125	0.1	6.25	6.25
176S	0.975	> 10	3.125	12.5	6.25
176R	0.122	0.15	0.2	3.125	6.25
208S	0.975	> 10	3.125	12.5	6.25
208R	0.122	0.15	0.2	12.5	6.25
CS01	0.487	>10	0.4	12.5	6.25
CR17	0.487	>10	0.4	12.5	6.25

## Discussion

In spite of the increased use of colistin for the treatment of *A. baumannii* infections, there are very few reports describing the emergence of colistin resistance due to complete loss of LPS biosynthesis through mutation of the genes involved in its biosynthesis [9,25-27]. Previous studies, and the results presented here, indicate that exposure to colistin can select for *A. baumannii* mutants that have high level colistin resistance due to loss of LPS production, presumably due to the absence of the molecule targeted by colistin on the bacterial membrane [8,9]. These studies, and data from the present study, also indicate that LPS loss can arise through a single mutational event, such as single base pair substitutions, deletions and insertions, and through gene interruption due to the insertion of transposable elements. Although highly speculative, in this context it may be expected that colistin resistance due to LPS loss would occur more frequently. One potential explanation for the low incidence of LPS-deficient *A. baumannii* may be that the loss of fitness associated with these mutations is sufficiently high so as to inhibit the stable emergence and subsequent transmission of these mutants. In order to address this issue, numerous traits associated with virulence were studied in colistin resistant, LPS-deficient *A. baumannii* strains.

We demonstrate that the loss of LPS is associated with a modest loss of fitness under laboratory conditions, and more dramatic loss of fitness under conditions that attempt to approximate human infection. A recent study by Beceiro *et al* has assessed the decrease in fitness and virulence of *A. baumannii* after loss of LPS expression [17]. In this study, similar to the results obtained in the present study, a modest decrease in bacterial fitness was observed in a mutant with a mutation in the *lpxA* gene when grown in laboratory medium (competition index 0.02). However the results obtained in rich medium, both in the study by Beceiro *et al*. and those obtained in the present study, are quantitatively very different than the competition index that we observed during *in vitro* growth in human serum (competition index 7.0 × 10^−7^). When using the results of the present study, these findings indicate that differences in growth medium can produce over 100,000-fold differences in observed competition indices between strains, highlighting the importance of the growth medium used when carrying out studies that compare fitness between bacterial strains. In the study reported by Beceiro *et al*. a disseminated mouse infection model was employed to compare the virulence [17]. In this model, the intraperitoneal administration of extremely high bacterial inocula are required for producing mortality (> 1 × 10^8^ CFU for the wild type ATCC 19606 strain). In the present study we attempted to mimic the clinical scenario in which a smaller bacterial inoculum (approximately 500-fold fewer bacteria) enters the body, resulting in bacterial growth and dissemination to distant organs. This model has previously been shown to result in bacterial dissemination and growth in distant organs [22], and has previously been employed for characterizing the fitness and virulence of colistin resistant *A. baumannii* strains with mutations in the PmrAB system [28,29]. In our model, LPS deficient *A. baumannii* was unable to survive and replicate *in vivo* (Fig. 2), and demonstrated a striking loss of fitness (competition index: 6.7 × 10^−8^). It is important to note that, although loss of LPS results in a striking loss of in vivo fitness and virulence, the mechanisms underlying this phenomenon are not clear. Changes in the ability to form biofilm and survive under iron limitation may play a role (discussed below). It is also possible that host factors play a role in this fitness reduction in light of a recent study demonstrating that LPS-deficient *A. baumannii* results in altered signaling through toll-like receptors and increased susceptibility to host antimicrobial peptides [16].

A recent study by our group evaluating the fitness of *A. baumannii* strains that acquire colistin resistance through mutation in the PmrAB system, which results in ethanolamine modification of LPS, showed that a colistin resistant derivative of the *A. baumannii* ATCC 19606 strain harboring mutations in the *pmrB* gene demonstrated a 62-fold decrease in *in vivo* fitness (competition index; 0.016) compared to its colistin susceptible parent strain [28]. In contrast, the decrease in fitness seen in the present study after loss of LPS production was over 1 million-fold higher (competition index: 6.7 × 10^−8^), indicating that, while LPS modifications due to mutations in the PmrAB system result in moderately lower fitness, the fitness decrease observed with complete loss of LPS may be substantially greater. In a separate study, the fitness of LPS deficient mutants and strains with mutations in pmrB were compared. LPS loss resulted in higher fitness costs than *pmrB* mutation in nutrient-rich medium, and antibiotic susceptibility testing results showed that the LPS-deficient but not the *pmrB* mutant had resistance profiles [18]. The Beceiro et al. study also demonstrated that LPS deficient mutants were highly attenuated in both C. elegans and mouse infection models, whereas a pmrB mutant was attenuated only in the C. elegans model [17]. Together, these studies may suggest that mutations in the PmrAB system result in a much less dramatic decrease in fitness compared to the loss of LPS under *in vivo* conditions.

In order to determine how LPS loss affects virulence traits that are potentially involved in pathogenesis and transmission of *A. baumannii*, biofilm formation, surface motility, growth under iron limitation and susceptibility to disinfectants were characterized in colistin resistant, LPS-deficient strains. Importantly, we previously demonstrated that loss of LPS can affect the phenotype of *A. baumannii* strains with respect to susceptibility to different antibiotics, including the phenomenon of colistin dependence [14]. Biofilm formation in *A. baumannii* is thought to play a role in antibiotic resistance, the establishment of infection and persistence on environmental surfaces [30,31]. The results presented here indicate that LPS loss results in decreased biofim formation compared to parental strains, suggesting that these strains may be defective in processes that require biofilm formation. With respect to surface motility, *A. baumannii* is able to move on semi-solid surfaces in response to environmental signals that are involved in bacterial virulence [1]. The role played by surface motility in bacterial virulence has not been elucidated, however it has recently been demonstrated that clinical isolates obtained from blood samples demonstrated more surface motility than isolates obtained for sputum, potentially suggesting a role for motility in certain infection types [32].

Bacterial pathogens experience iron limitation both in the environment and during host infection, suggesting that survival under iron limitation may contribute to pathogenesis and transmission of bacterial pathogens [33]. The results presented here indicate that LPS loss results in a severe growth defect under iron limitation, as no growth was observed in low iron conditions for any of the six LPS-deficient strains tested. These findings may, at least in part, explain the lack of growth seen in human serum (Fig. 1B) and in vivo (Fig. 2) in the present study. Although these findings associate LPS loss with reduced growth under iron limitation, the molecular mechanism underlying this finding is not yet apparent. One possibility is that the altered membrane composition that has been described in LPS deficient *A. baumannii* [19], results in a reduced ability to import extracellular iron, although this has not been tested experimentally. It is of note that a previous study by our group demonstrated reduced growth under iron limiting conditions in a strain acquiring colistin resistance through mutations in the PmrAB system, which is consistent with the result seen here with strains CS01 and CR17 [34].

The ability of bacteria to survive in clinical environments may facilitate to their transmission. In this context, resistance to commonly used disinfectants may contribute to the spread of pathogens such as *A. baumannii*, which are known to persist on hospital surfaces. The data presented here indicate that LPS loss produces a marked increase in susceptibility to certain disinfectants that are commonly used in the hospital setting, specifically to chlorhexidine, deoxycholate and sodium dodecyl sulfate. This may indicate that LPS-deficient strains are less able to persist in the context of hygiene procedures that employ these agents in disinfection protocols, thus decreasing the transmissibility of these strains in clinical settings.

The results presented here also indicate that mutations in the PmrAB system (strains CS01 and CR17) can affect biofilm formation, surface motility and growth under iron limitation, but not susceptibility to different disinfectants. Our group and others have previously demonstrated that *A. baumannii* strains with mutations in the PmrAB system (including strains CS01 and CR17 used in the present study) demonstrate reduced fitness and virulence [28,35-38]. It is important to note, however, that separate studies assessing fitness in PmrAB mutant strains demonstrated no changes in fitness compared to parental strains [35,39,40]. It has been suggested that these differences may be due to the presence of compensatory mutations or differences in virulence of the parental strains used in the studies [35]. A recent study evaluating two A. baumannii clinical isolates that acquired mutations in the *pmrB* gene during colistin treatment demonstrated that these stains had reduced growth rates in vitro [41]. Interestingly, these colistin resistant strains were associated with less invasive infection in patients compared to their colistin susceptible counterparts. While the underlying molecular mechanisms that lead to these changes in strains with mutations in the PmrAB system, a proteomic study comparing a strain with a mutation in *pmrA* with its wild type counterpart indicated that 35 proteins were differentially expressed, including outer membrane proteins, chaperones, protein biosynthesis factors, and metabolic enzymes [42]. These results may indicate that mutations in the PmrAB system have broad effects on the physiology of *A. baumannii*.

In summary, the work presented here demonstrates the ability of *A. baumannii* to adapt to antibiotic pressure through loss of a major virulence factor and structural component, even though this adaptation results in a dramatic decrease in fitness. It is important to point out that, although this study associates loss of LPS with multiple phenotypic changes, the underlying molecular mechanisms that produce these changes remain unknown. Recent studies indicating that the expression of numerous genes and the multiple metabolic functions are affected in strains lacking LPS suggest global changes in the physiology of LPS-deficient strains compared to their LPS-replete counterparts [19,43]. The findings presented here establish a link between the acquisition of colistin resistance through LPS loss and decreased fitness and virulence, which may provide insights into the low prevalence of colistin resistant isolates with LPS loss in the clinical setting.
